# Chemotherapy-Induced Brain Damage: Mechanisms and Insights from Rodent Models

**DOI:** 10.3390/brainsci16070750

**Published:** 2026-07-15

**Authors:** Milica Veljković, Tanja Džopalić, Pavle Ranđelović, Lidija Popović Dragonjić, Jelena Milenković, Ivan Ilić

**Affiliations:** 1 Department of Physiology, Medical Faculty, University of Niš, Zoran Đinđić Boulevard 81, 18 000 Niš, Serbia; pavleus@gmail.com; 2 Department of Immunology, Medical Faculty, University of Niš, Zoran Đinđić Boulevard 81, 18 000 Niš, Serbia; tanja.dzopalic@medfak.ni.ac.rs; 3 Department of Infectious Diseases and Epidemiology, Medical Faculty, University of Niš, 18 000 Niš, Serbia; lidija_popovic2003@yahoo.com; 4 Clinic for Infectology, University Clinical Center Niš, 18 000 Niš, Serbia; 5 Department of Pathological Physiology, Medical Faculty, University of Niš, Zoran Đinđić Boulevard 81, 18 000 Niš, Serbia; jelena.milenkovic@medfak.ni.ac.rs; 6 Department of Pathology, Medical Faculty, University of Niš, Zoran Đinđić Boulevard 81, 18 000 Niš, Serbia; ilicko81@gmail.com; 7 Clinic for Pathology, University Clinical Center Niš, 18 000 Niš, Serbia

**Keywords:** chemobrain, rodent models, chemotherapeutic agents, cognitive deficits, neurotoxicity, behavioral outcomes

## Abstract

Background/Objectives: Chemotherapy-induced cognitive impairment, colloquially known as chemobrain, affects a substantial proportion of cancer patients. Preclinical rodent models help clarify underlying drug-specific neurotoxic effects, as well as histological, biochemical, molecular, and behavioral mechanisms. Methods: We conducted a narrative review of animal studies examining cognitive dysfunction following treatment with commonly used chemotherapeutic agents, including doxorubicin, cisplatin, cyclophosphamide, methotrexate, 5-fluorouracil, paclitaxel, and docetaxel. The review focused on behavioral and cognitive outcomes, and experimental parameters such as rodent models and dosing regimens. Results: Across studies, chemotherapeutic exposure has had a consistent negative effect on short-term and working memory, learning and other cognitive domains, with impairments being often mild and detectable even at doses not causing apparent systemic toxicity. Histological analyses revealed reduced neurogenesis, dendritic and myelin alterations, and glial activation, mainly in the hippocampus and prefrontal cortex. Biochemical and molecular changes included oxidative stress, pro-apoptotic signaling, inflammatory cytokine dysregulation, decreased neurotrophic support, and altered neurotransmitter dynamics. Age and sex influenced susceptibility, with juvenile or aged animals and females—particularly older females modeling breast cancer patients—showing greater deficits. Cumulative or repeated dosing exacerbated neurotoxicity, while single administrations produced milder, sometimes transient, impairments. Conclusions: Preclinical models provide compelling evidence that chemotherapeutic agents impair cognitive function via convergent mechanisms involving inflammation, oxidative stress, and synaptic dysregulation. These findings highlight the importance of considering age, sex, and treatment schedule in designing neuroprotective strategies and underscore the translational relevance of rodent models in understanding chemobrain.

## 1. Introduction

Chemotherapy has significantly improved survival rates for many cancer patients, yet it is increasingly recognized that systemic cancer treatments can have unintended effects on the central nervous system [1,2]. Among these intense, occasionally enduring or irreversible side effects, chemotherapy-induced cognitive impairment, commonly referred to as “chemobrain,” has emerged as a major concern [3]. Short-term memory, working memory, and verbal abilities are typically the most vulnerable, with visuospatial skills, executive functioning, and attention span also showing a measurable decline [2,3]. Since these impairments are often mild, affected cancer survivors usually remain within the normal performance range, although consistently near its lower limits [3,4]. Additionally, the impairments may persist long after the completion of therapy and negatively impact daily functioning and quality of life [5]. As a consequence, affected individuals often experience difficulties resuming professional or academic activities, maintaining social relationships, and fulfilling everyday family and caregiving responsibilities [6]. Despite its prevalence, the mechanisms underlying these cognitive deficits are not yet fully understood [3,7], highlighting the need for preclinical studies.

Research has shown a reduction in gray matter density in multiple brain regions in chemobrain, including the frontal and temporal cortices, with only partial restoration observed after one year [8,9]. In contrast, some studies report increased activity in certain regions, suggesting a compensatory response by means of which cancer survivors recruit additional cognitive resources to perform the same tasks. As a consequence, these resources are exhausted more rapidly during more demanding cognitive activities [10,11].

Animal models have proven to be invaluable tools for investigating the neurotoxic effects of chemotherapy. Rodent models, in particular, offer controlled environments in which researchers can explore cellular, molecular, and behavioral alterations following treatment. These models allow a detailed examination of histopathological changes, oxidative stress, neuroinflammation, and alterations in neurogenesis that are difficult or impossible to study directly in humans. Moreover, animal studies provide a platform for testing interventions aimed at mitigating cognitive side effects, thus facilitating the development of strategies that could improve patient outcomes [1].

Various chemotherapeutic agents, including anthracyclines, platinum-based compounds, antimetabolites, and taxanes, have been shown to induce neurotoxicity in preclinical models. Through a structured overview, this work aims to enhance the understanding of chemobrain pathophysiology and to highlight potential approaches for future preclinical and translational research. The review specifically focuses on rodent models (mice and rats) and provides an updated and structured synthesis of behavioral, cognitive, and mechanistic findings, emphasizing recent advances and converging pathways across different chemotherapeutic agents.

## 2. Literature Search Strategy

A comprehensive literature search was conducted to identify studies on rodent models of chemotherapy-induced brain damage. The following electronic databases were searched: PubMed, Scopus, and Web of Science. Additionally, Google Scholar was consulted to identify any additional relevant articles.

Initially, a large body of literature was identified and reviewed (>100 studies). Following the removal of duplicates, titles and abstracts were screened, and full texts were evaluated, resulting in the final set of studies included in this review.

Keywords and search strings included combinations of terms such as “chemotherapy”, “brain damage”, “neurotoxicity”, “rodent models”, and “cognitive impairment”. The search was limited to studies published between January 2010 and March 2026. Inclusion criteria were: original research articles and relevant review articles involving rodent models of chemotherapy-induced neurotoxicity, studies reporting behavioral, histological, or molecular outcomes, and publications in English. Exclusion criteria were studies on humans and studies without relevant neurotoxic outcomes. Clinical studies were excluded from the systematic search; however, relevant human data were considered in the discussion to support mechanistic interpretation and translational relevance.

The chemotherapeutic agents included in this review were selected on the basis of clinical relevance, prevalence of reported cognitive and neurotoxic effects, as well as the availability of sufficient preclinical and clinical data. While other agents, such as vincristine and vinblastine, are mentioned in the mechanistic sections due to their known neurotoxic potential, they were not included in the full narrative review as detailed evidence on cognitive outcomes was limited or inconsistent. This approach ensured that the review focused on agents for which robust experimental and behavioral data were available, allowing meaningful synthesis and comparison across studies.

In addition, ethical considerations related to the use of animal models in the reviewed studies should be acknowledged. Greater emphasis on the principles of replacement, reduction, and refinement (3Rs), along with the development of alternative models, may improve both ethical standards and translational relevance.

## 3. General Mechanisms of Chemotherapy-Induced Brain Damage

### 3.1. Neuroinflammation

Although different chemotherapeutic agents vary in their primary anticancer mechanisms, many of them trigger similar pathogenic processes within the central nervous system. A central feature of chemotherapy-related neurotoxicity is the induction of neuroinflammation [2], characterized by activation of microglia and astrocytes, increased production of pro-inflammatory cytokines, and disruption of normal glial-neuronal communication. These inflammatory changes can alter synaptic plasticity and contribute to long-lasting cognitive deficits. Since glial cells undergo ongoing proliferation and turnover throughout life, they are especially sensitive to chemotherapeutic exposure. Damage affecting either neurons or glial population may subsequently activate microglia and astrocytes, initiating neuroinflammatory responses that play a role in persistent functional deficits [3].

### 3.2. Cellular Senescence

Recent evidence suggests that chemotherapy may also induce cellular senescence in neural and glial cells, contributing to persistent neuroinflammation through the release of pro-inflammatory mediators. This mechanism may further exacerbate neuropathic symptoms [12,13].

### 3.3. Microbiome-Brain Axis

Evidence also indicates that chemotherapy may disrupt the gut microbiome, which in turn can influence the gut–brain axis and contribute to neuroinflammation and neuropathic symptoms. Alterations in microbial composition have been associated with changes in immune signaling and central nervous system function, representing a potential mechanism underlying chemobrain [14,15].

### 3.4. Neuroimaging Biomarkers

Additionally, neuroimaging studies in clinical populations have identified structural and functional brain changes associated with chemotherapy exposure, supporting the concept of central nervous system involvement in chemobrain. These findings suggest that neuroimaging biomarkers may provide valuable insight into the neural correlates of chemotherapy-induced neurotoxicity [16].

### 3.5. Glial Cell Changes and Blood–Brain Barrier (BBB)

Reduced gliogenesis in adult neurogenic regions, such as the hippocampus and lateral ventricles, may result in a diminished generation of new astrocytes and oligodendrocytes. As these glial populations actively participate in memory encoding, consolidation [17,18], and information processing, their depletion can negatively affect cognitive function. In particular, proper oligodendrocyte turnover is essential for axonal myelination, which supports efficient signal conduction and cognitive performance across the lifespan [19,20,21]. Astrocytes play a central role in neuroinflammation by responding to pro-inflammatory cytokines. During astrogliosis, astrocyte activity shifts from protective to detrimental, leading to excessive release of inflammatory signals that drive oxidative and nitrosative stress and impair neuroplasticity. Elevated GFAP levels are a hallmark of this reactive state [5]. Experimental studies further suggest that oligodendrocyte precursor cells (OPC), as well as mature non-dividing oligodendrocytes, are most notably vulnerable to chemotherapy, potentially leading to long-term cognitive and motor deficits [22,23,24,25].

Activated microglia and astrocytes release cytokines within the CNS, which can modulate neuronal function. In addition, cytokines originating from peripheral tissues (such as IL-1B, IL-6 and TNF-α) can penetrate the brain and further stimulate local cytokine production [6,26,27]. These inflammatory mediators may compromise the integrity of the blood–brain barrier (BBB), facilitating the entry of additional cytokines and, potentially, chemotherapeutic agents. Elevated levels of inflammatory cytokines have been linked to impaired cognitive performance in both preclinical and clinical studies [28].

### 3.6. Oxidative Stress

Another commonly implicated mechanism is oxidative stress [2,29]. Several cytotoxic agents, such as doxorubicin, cyclophosphamide, and methotrexate, induce the generation of reactive oxygen species (ROS), overwhelm endogenous antioxidant defenses, and damage cellular structures, including lipids, proteins, and nucleic acids [1,29]. Oxidative injury is closely linked to mitochondrial dysfunction, which further compromises neuronal energy metabolism and increases susceptibility to apoptotic pathways [1].

### 3.7. DNA Changes

The postmitotic brain accounts for only a small fraction of body weight, yet it consumes a disproportionately large amount of energy [30], leading to high levels of ROS and increased risk of DNA damage. Cancer predominantly affects older adults, with half of new cases diagnosed between ages of 54 and 74 [31]. Combined with additional stressors—such as the tumor itself and psychiatric conditions like depression—this creates a particularly vulnerable environment for the brain [3].

Chemotherapeutic agents, especially those that target DNA, can induce genomic injury in postmitotic neurons, which in turn promotes premature cellular aging and ultimately leads to neuronal loss [32]. Because the mature brain has a limited capacity for DNA repair, the accumulation of chemotherapy-related damage may further accelerate neuronal dysfunction and degeneration [3].

### 3.8. Microtubule Changes

Several chemotherapeutic agents exert neurotoxic effects through microtubule-related mechanisms [33]. They interfere with microtubule dynamics by either inducing excessive stabilization, as observed with paclitaxel and docetaxel, or promoting microtubule disassembly, as seen with agents such as vincristine and vinblastine. Microtubules play a crucial role in maintaining neuronal polarity and morphology, supporting axonal transport, and organizing intracellular signaling platforms [34]. Consequently, both abnormal stabilization and destabilization of microtubules can impair neuronal structure, function, and intracellular communication [3].

### 3.9. Neurogenesis

Some chemotherapeutic agents can also affect neurogenesis, particularly within the subgranular zone (SGZ) of the dentate gyrus of the hippocampus, a region essential for learning and memory. Other areas of adult neurogenesis that may be affected include the subventricular zone (SVZ) lining the lateral ventricles [35] and the striatum [36]. Under normal conditions, neurogenesis compensates for the neurons lost through daily activity [37]. After acute challenges—such as stroke or chemobrain—neurogenesis plays a key role in supporting cognitive recovery [38]. Reduced proliferation of neural precursor cells, impaired maturation of newborn neurons, and altered dendritic morphology have been documented in multiple preclinical studies. These changes are thought to add to deficits in memory consolidation and spatial learning. Chemotherapy has been associated with reductions in several protein markers of neurogenesis, though findings are not entirely consistent. These include BrdU and Ki-67, which mark proliferating cells; doublecortin (DCX), present in neural progenitor cells and immature neurons; and NeuN, indicative of mature neurons [39]. Brain-derived neurotrophic factor (BDNF) belongs to the neurotrophin family and supports neurogenesis by promoting the survival, growth, and differentiation of new neurons. Clinical studies have linked reduced serum BDNF levels with cognitive deficits in cancer patients, highlighting its potential role in chemotherapy-associated cognitive impairment [40,41].

Dendritic spines and arborization not only regulate synaptic plasticity, which underlies learning, memory, and executive functions [42], but also continuously proliferate and remodel in response to experience [42]. Loss or simplification of these structures can lead to cortical thinning, potentially contributing to the reductions in gray matter observed in cancer survivors after chemotherapy [3]. Administration of cisplatin [43], 5-fluorouracil [44], doxorubicin, and cyclophosphamide has been associated with decreased dendritic complexity and spine density in hippocampal granule cells and pyramidal neurons of the CA1 and CA3 regions [45,46].

### 3.10. Neurotransmitter Changes

Furthermore, chemotherapy can influence neurotransmitter systems, including glutamatergic, dopaminergic, and cholinergic signaling. Altered neurotransmission can disrupt network connectivity and play a role in behavioral and cognitive abnormalities [3]. Although these mechanisms do not occur uniformly across all drugs, they provide a conceptual framework for understanding how various chemotherapeutic agents may converge to produce brain injury. Genetic studies implicating catechol-O-methyltransferase (COMT)—a key enzyme regulating dopamine, epinephrine, and norepinephrine metabolism—provide additional support for the involvement of neurotransmitter systems in chemotherapy-associated cognitive impairment [40]. Variants associated with increased COMT activity, and consequently reduced cortical dopamine availability, have been linked to a higher risk of developing chemobrain in cancer survivors [47,48]. This suggests that limited dopaminergic reserve may increase vulnerability to chemotherapy-induced cognitive deficits.

## 4. Animal Models and Experimental Approaches

Preclinical research on chemotherapy-related neurotoxicity relies on various animal models and experimental strategies that approximate the cognitive and neural changes observed in patients. No single model captures the full complexity of the human brain; however, combining species, age groups, and diverse analytical methods provides a reliable framework for investigating the biological processes underlying chemotherapy-induced brain injury. Many chemobrain animal models are tumor-free, which may underestimate the cognitive and neuroinflammatory effects observed in human patients, since the presence of tumor itself can contribute to these impairments [1,6,49].

### 4.1. Rodent Models: Mice and Rats

Mice and rats are the most commonly used animal models in preclinical research on chemotherapy-induced cognitive impairment. Their well-characterized neuroanatomy, the availability of genetically modified strains (particularly in mice), and the ease of maintaining laboratory colonies make them highly suitable for mechanistic and behavioral studies. While mice are frequently used to investigate molecular and cellular pathways, rats are often preferred for complex behavioral assessments due to their larger brain size, which facilitates detailed histological and neurobehavioral analyses [1].

This review focuses on rodent models (mice and rats) and examines behavioral and cognitive outcomes following chemotherapy.

### 4.2. Age-Dependent Susceptibility

Animal age significantly affects vulnerability to neurotoxic effects. Most studies focus on adult rodents (8–12 weeks old) to model adult patients. However, increasing attention is being given to juvenile and adolescent models, reflecting the use of chemotherapeutic agents in pediatric oncology. Younger animals often show higher susceptibility due to ongoing neurogenesis, immature synaptic connections, and a developing blood–brain barrier. Conversely, some studies use older rodents to model age-associated cognitive decline or to investigate whether chemotherapy accelerates aging processes [50]. Studies on paclitaxel-treated rodents have shown that both juvenile and aged animals develop more severe neuropathic symptoms compared with young adults [51]. Age-related differences are also evident at the cellular level, where alterations in neuronal structure and regenerative capacity may contribute to increased neurotoxicity [52]. Nevertheless, most preclinical studies are conducted on young adult rodents, corresponding approximately to early adulthood in humans [51], while research on juvenile or aged populations remains limited [53,54]. This represents a notable gap, particularly in light of the clinical relevance of pediatric and older patient populations [55].

### 4.3. Sex-Dependent Differences

Sex-related differences in neurotoxicity are increasingly being recognized. Hormones such as estrogen and androgens influence neuroinflammatory pathways, oxidative stress responses, and neurogenesis, which can modify the severity and pattern of cognitive impairments. Incorporating both male and female animals allows researchers to identify sex-specific vulnerabilities and potential therapeutic targets. Given that most breast cancer patients are aged 60 or older at diagnosis, older female rodents are often used in preclinical studies to better model the clinical population [50]. In paclitaxel-based models, female rodents have, in some cases, exhibited more pronounced or prolonged mechanical allodynia compared with males [56,57], although opposite findings have also been reported [58], indicating variability across experimental conditions. Importantly, sex-specific differences extend to pharmacological responses, as certain neuroprotective interventions have shown efficacy in males but not in females. Despite this, the majority of preclinical chemobrain studies (approximately 70–85%) rely exclusively on male animals, limiting the translational relevance of these findings [59,60]. This imbalance is particularly significant given the clinical predominance of female patients in certain cancer populations, underscoring the need for more systematic inclusion of both sexes in experimental designs [55].

### 4.4. Routes and Schedules of Chemotherapy Administration

Chemotherapeutic agents are administered through various routes—intraperitoneal (i.p.), intravenous (i.v.), or oral—with dosing regimens designed to mimic clinical schedules while minimizing systemic toxicity [6]. Single versus repeated dosing paradigms are selected according to the study objectives, with repeated exposure typically providing a more clinically relevant model of cumulative neurotoxic effects.

### 4.5. Behavioral Assays

Behavioral testing is essential for evaluating cognitive and emotional outcomes. The Morris Water Maze (MWM) and the Barnes Maze (BM) are commonly employed for spatial learning and memory, while the Novel Object Recognition test (NOR) assesses recognition memory [6]. The Open Field test (OFT) evaluates locomotor activity and anxiety-like behaviors. In addition to memory-based tasks, attention, impulsivity, and executive function are commonly assessed using paradigms such as the five-choice serial reaction time task (5CSRTT) [6]. Additional specialized approaches, including fear conditioning and operant learning tasks, can further examine specific cognitive domains in greater detail.

### 4.6. Histological and Molecular Assessments

Histological analyses focus on neuronal loss, dendritic morphology, synaptic integrity, and glial activation. Molecular approaches measure oxidative stress markers, inflammatory cytokines, apoptotic pathways, and neurotrophic factors. Together, these evaluations provide a comprehensive link between observed behavioral deficits and underlying cellular and molecular alterations.

## 5. Limitations and Translational Challenges of Rodent Models

A key limitation of current preclinical studies on chemotherapy-induced brain damage is the use of tumor-free rodent models, which may not fully replicate the clinical context of cancer patients. Moreover, behavioral endpoints in rodents do not always correspond to human cognitive syndromes, highlighting the challenge of translational relevance. Variability in dosing regimens and routes of administration further complicates comparison across studies, while differences between species and strains can affect the generalizability of findings. By acknowledging these factors, we aim to provide a balanced view of the strengths and limitations of the available preclinical evidence.

Although the majority of preclinical studies in our review employed tumor-free animals, we also included and discussed selected studies using tumor-bearing models. These studies demonstrate that the presence of a tumor can influence both neuroinflammatory responses and behavioral outcomes, emphasizing the added translational relevance of tumor-bearing models for understanding chemotherapy-induced cognitive dysfunction.

In one study, following the injection of human metastatic breast cancer cells into mice, subsequent chemotherapy treatment with paclitaxel, docetaxel, or eribulin led to increased tracer and drug accumulation in the brain, alongside elevated astrocyte activation, indicating enhanced neuroinflammatory responses [61]. A second study employed a comprehensive post-menopausal breast cancer model in immunocompetent ovariectomized mice, combining orthotopic tumors, chemotherapy, and hormone therapy, which revealed cumulative effects on neuroinflammation, locomotor activity, and brain microstructure [62]. These findings support the relevance of tumor-bearing models in capturing additional neurotoxic and behavioral consequences of chemotherapy, providing a more translationally relevant perspective on chemobrain.

## 6. Chemotherapeutic Agents and Cognitive Impairment

### 6.1. Overview of Major Chemotherapeutic Agents

#### 6.1.1. Doxorubicin

Doxorubicin is an anthracycline chemotherapeutic agent widely used in the treatment of both hematological malignancies and solid tumors, including ovarian, prostate, thyroid cancers, and Hodgkin’s disease. Its anti-cancer effects are mediated through DNA intercalation, oxidative stress and inhibition of topoisomerase II, leading to apoptosis in rapidly dividing cells. Despite its therapeutic efficacy, doxorubicin damages healthy tissues throughout the body, causing cardiotoxicity, hepatotoxicity, and nephrotoxicity [1,27]. Preclinical studies in rodents are therefore critical to investigate potential neurotoxic effects, providing insight into the mechanisms associated with chemotherapy-induced cognitive and behavioral impairments.

#### 6.1.2. Cisplatin

Cisplatin is a platinum-based chemotherapeutic agent commonly used to treat testicular, ovarian, head and neck, bladder, cervical, and esophageal tumors, as well as small cell lung carcinoma (SCLC). Its cytotoxic effect is primarily driven by the formation of DNA crosslinks, which initiate apoptosis in rapidly dividing tumor cells. Although clinically effective, its usage is limited because of significant off-target toxicities, including neurotoxicity, nephrotoxicity, ototoxicity, cardiotoxicity, hepatotoxicity, and gastrotoxicity [63], often necessitating careful management of dose and treatment schedule. Preclinical studies in rodents have been crucial in clarifying the neurotoxic mechanisms responsible for cisplatin-induced cognitive deficits [1,64].

#### 6.1.3. Cyclophosphamide

Cyclophosphamide is an alkylating chemotherapeutic agent widely used for treating hematological malignancies and solid tumors, including breast, ovarian, and lymphoid cancers. It exerts its anti-cancer effects by forming DNA crosslinks, leading to impaired DNA replication and apoptosis in rapidly dividing tumor cells. Despite being effective, cyclophosphamide is associated with systemic toxicities, such as myelosuppression, hemorrhagic cystitis, and neurotoxicity, which can affect dosing regimens and treatment duration. Preclinical rodent models have been widely employed to study cyclophosphamide-induced neurotoxic effects and to explain the molecular, cellular, and behavioral mechanisms underlying chemotherapy-related cognitive impairments [1,65].

#### 6.1.4. Methotrexate

Methotrexate is an antimetabolite chemotherapeutic agent commonly used in the treatment of leukemia, lymphoma, and various solid tumors, including breast, head and neck, and osteosarcoma. It produces its anticancer effects primarily by inhibiting dihydrofolate reductase, thus blocking DNA synthesis and cell proliferation in rapidly dividing tumor cells. Though therapeutically effective, methotrexate is associated with adverse effects, including hepatotoxicity, nephrotoxicity, and neurotoxicity, which may influence treatment regimens. Preclinical rodent models have been widely used to investigate methotrexate-induced neurotoxic effects and the core molecular and cellular mechanisms underlying cognitive impairments [1,66].

#### 6.1.5. 5-Fluorouracil (5-FU)

5-Fluorouracil is a pyrimidine analog chemotherapeutic agent widely used to treat colorectal, breast, and gastrointestinal cancers. It exerts its anti-cancer activity by inhibiting thymidylate synthase and incorporating into RNA and DNA, thus disrupting nucleic acid metabolism and impairing the proliferation of rapidly dividing tumor cells. Despite its clinical efficacy, 5-FU is associated with systemic toxicities, including myelosuppression, gastrointestinal toxicity, and neurotoxicity, which can influence dosing schedules and treatment duration. Preclinical rodent studies have been instrumental in investigating the neurotoxic effects of 5-FU and the fundamental cellular and molecular mechanisms driving chemotherapy-induced cognitive impairments [1,65].

#### 6.1.6. Paclitaxel

Paclitaxel is a taxane chemotherapeutic agent commonly used for treating breast, ovarian, and lung cancers. It stabilizes microtubules and prevents their depolymerization, thereby disrupting mitotic spindle formation and inhibiting cell division in rapidly proliferating tumor cells. While highly effective against cancer, paclitaxel can also induce off-target toxicities, including peripheral neuropathy and neurocognitive impairments, which may influence treatment schedules. Rodent models have proven valuable in studying paclitaxel-induced neurotoxicity, allowing investigation of the underlying cellular, molecular, and behavioral mechanisms [1,62,65].

#### 6.1.7. Docetaxel

Docetaxel is a semisynthetic taxane chemotherapeutic agent widely used in the treatment of breast, prostate, and non-small cell lung cancers. It acts through the stabilization of microtubules, thus interfering with mitotic spindle dynamics and inhibiting cell division in rapidly proliferating tumor cells. Despite its potent anti-cancer effects, its use is limited by side effects including peripheral neuropathy and cognitive impairments, which can affect dosing and treatment strategies. Preclinical studies in rodent models have been essential for exploring the neurotoxic potential of docetaxel and understanding molecular, cellular, and behavioral mechanisms responsible for chemotherapy-related cognitive deficits [1,65].

### 6.2. Mechanisms of Neurotoxicity Across Chemotherapeutic Agents

#### 6.2.1. Oxidative Stress and Mitochondrial Dysfunction

Oxidative stress represents a central mechanism in chemotherapy-induced neurotoxicity, involving excessive reactive oxygen species (ROS) production, mitochondrial impairment, and subsequent activation of apoptotic pathways.

Doxorubicin induces ROS generation through redox cycling, leading to oxidative stress in neuronal and glial cells and contributing to disrupted cellular homeostasis [67]. Similarly, cisplatin promotes mitochondrial dysfunction and excessive ROS production, triggering intrinsic apoptotic signaling and neuronal injury [64,68]. Cyclophosphamide also exerts neurotoxic effects via induction of oxidative stress and downstream apoptotic activation [65], while methotrexate disrupts mitochondrial function secondary to impaired folate metabolism, leading to oxidative imbalance and neuronal apoptosis [69].

5-Fluorouracil further contributes to oxidative stress through inhibition of thymidylate synthase, resulting in mitochondrial dysfunction and neuronal damage [65]. Taxanes, including paclitaxel and docetaxel, impair mitochondrial integrity through disrupted axonal transport and increased ROS production [62,65,70], ultimately promoting neuronal apoptosis.

#### 6.2.2. Neuroinflammation and Glial Activation

Neuroinflammatory processes and glial activation are consistently observed across chemotherapeutic agents and represent a major contributor to cognitive impairment.

Doxorubicin increases peripheral and central pro-inflammatory cytokines, including TNF-α, which can cross the blood–brain barrier and activate inflammatory cascades in the CNS [67]. Cisplatin also elevates circulating inflammatory cytokines, promoting glial activation and linking systemic inflammation to cognitive dysfunction [71].

Cyclophosphamide enhances microglial activation and increases hippocampal cytokine expression, indicating a clear neuroinflammatory component to its toxicity [67,72]. Methotrexate induces sustained microglial activation and widespread glial dysregulation involving astrocytes and oligodendrocytes, contributing to impaired myelination and neuronal dysfunction [69,73,74]. Although CNS cytokine levels may not always increase significantly, peripheral inflammation may still contribute to CNS effects [71].

5-Fluorouracil increases hippocampal cytokine levels and contributes to neuroinflammatory signaling associated with cognitive deficits [44,67]. Paclitaxel and docetaxel both increase peripheral inflammatory mediators and are associated with microglial and astrocytic activation in hippocampal regions, further linking neuroinflammation to chemotherapy-induced cognitive impairment [67,71,75,76,77].

#### 6.2.3. Blood–Brain Barrier and Vascular Dysfunction

Several chemotherapeutic agents exert direct or indirect effects on the blood–brain barrier (BBB) and cerebral vasculature, contributing to increased CNS vulnerability.

Doxorubicin exerts neurotoxic effects predominantly through indirect mechanisms, despite limited BBB penetration [67]. Cisplatin may influence the CNS indirectly through systemic inflammation and cytokine-mediated signaling [71].

Methotrexate may impair cerebral blood flow and glucose metabolism, while also exerting antiangiogenic effects that compromise vascular integrity [78]. Paclitaxel induces endothelial senescence, reduces microvascular density, and disrupts neurovascular coupling, thereby impairing BBB integrity and increasing CNS susceptibility to circulating toxic mediators [79].

#### 6.2.4. DNA Damage and Apoptotic Signaling

Direct DNA damage and activation of apoptotic pathways represent key mechanisms for several alkylating and platinum-based agents.

Cisplatin induces intra- and interstrand DNA crosslinks, leading to transcriptional arrest and genomic instability in postmitotic neurons, ultimately triggering apoptosis [64,68]. Cyclophosphamide produces DNA alkylation, contributing to neuronal dysfunction and cell death [65]. Doxorubicin inhibits topoisomerase IIβ activity, leading to DNA damage and activation of apoptotic cascades [80].

Methotrexate indirectly contributes to neuronal apoptosis through metabolic disruption and impaired nucleotide synthesis [69], while 5-Fluorouracil interferes with DNA and RNA synthesis, ultimately promoting apoptotic neuronal loss [65].

Taxanes such as paclitaxel and docetaxel also activate apoptotic signaling secondary to microtubule disruption and axonal transport failure [62,65,70].

#### 6.2.5. Neurotrophic Signaling and Neurogenesis Impairment

A consistent finding across chemotherapeutic agents is disruption of neurotrophic support and impaired neurogenesis.

Doxorubicin reduces hippocampal BDNF levels, suggesting impaired neurotrophic signaling and reduced neurogenesis [80]. Methotrexate similarly decreases cortical BDNF expression and disrupts glial–neuronal interactions that support myelination and neuronal survival [3,69,74]. 5-Fluorouracil also reduces hippocampal BDNF levels, contributing to impaired learning and memory [81].

Collectively, these alterations suggest that reduced neurotrophic support is a shared pathway contributing to chemotherapy-related cognitive impairment.

#### 6.2.6. Neurotransmitter and Signaling Dysregulation

Methotrexate may impair neurotransmitter balance and excitotoxic regulation, while paclitaxel affects calcium signaling pathways and synaptic stability [69,75]. These effects further amplify neuronal vulnerability and contribute to long-term cognitive dysfunction.

### 6.3. Histological Changes in Rodent Models Across Chemotherapeutic Agents

Rodent studies consistently demonstrate that chemotherapeutic agents induce structural alterations in brain regions critical for cognition, particularly the hippocampus and prefrontal cortex. Common histopathological findings include neuronal loss, reduced dendritic complexity and arborization, dendritic spine loss, synaptic alterations, and impaired neuronal connectivity. These changes have been reported following treatment with doxorubicin, cisplatin, cyclophosphamide, methotrexate, 5-Fluorouracil (5-FU), paclitaxel, and docetaxel, although their severity and regional distribution vary among agents.

Neuronal degeneration is accompanied by impaired neurogenesis in several experimental models. Doxorubicin treatment did not alter the number of BrdU-positive proliferating cells but reduced DCX-positive and BrdU/NeuN double-labeled cells [72], indicating impairment of both early and late stages of neuronal maturation. Cyclophosphamide produced a similar pattern, sparing BrdU-positive proliferating cells while decreasing DCX-positive and BrdU/NeuN double-labeled cells [72]. Likewise, 5-FU treatment did not affect Ki-67-positive proliferating cells but reduced DCX-positive cells [81]. In addition, 5-FU decreased proliferation of neural precursor cells in the hippocampal SGZ, dentate gyrus, the SVZ [78], and the corpus callosum [23]. Similarly, systemic cisplatin exposure decreased cell proliferation in the hippocampal SGZ, the SVZ lining the lateral ventricles, and the corpus callosum [22], whereas paclitaxel reduced hippocampal neurogenesis, as evidenced by decreased numbers of Ki67- and BrdU-positive cells in the subgranular zone [1,2,82].

Several chemotherapeutic agents also induce glial alterations indicative of neuroinflammation. Doxorubicin significantly increased astrocyte reactivity in the hippocampus, as evidenced by elevated glial fibrillary acidic protein (GFAP)-positive cell number [5], signal intensity, and cellular volume in treated rats compared with controls [83]. Histopathological studies of cisplatin similarly reported evidence of gliosis, suggesting an accompanying neuroinflammatory response that may further increase neuronal damage [64,84]. Methotrexate induced region- and time-dependent glial alterations, with increased GFAP expression observed in the hippocampus and prefrontal cortex at acute (96 h) and subacute (31 days) time points. However, no significant elevation in GFAP expression was detected at the chronic time point (93 days), suggesting that methotrexate-induced neuroinflammation may be transient [6]. In contrast, 5-FU treatment induced pronounced astrocytic reactivity in both the hippocampus and prefrontal cortex during acute and subacute stages, while persistent GFAP elevation remained detectable in the hippocampus at chronic time points [6], indicating region-specific long-lasting neuroinflammatory changes.

In addition to these common histopathological features, several agent-specific structural alterations have also been reported. Doxorubicin caused increased numbers of apoptotic cells in regions vulnerable to oxidative stress, whereas massive neuronal loss was not consistently observed. Cisplatin reduced dendritic spine density and arborization in the cingulate cortex [64,84]. Methotrexate additionally disrupted white matter architecture and myelin integrity, suggesting treatment-associated demyelination and impaired axonal signaling [6]. Paclitaxel induced axonal degeneration and microtubule disorganization, together with reductions in dendritic arborization and spine density, while docetaxel likewise caused microtubule disorganization and synaptic alterations associated with cognitive impairment [1,2,65,82,85].

### 6.4. Biochemical and Molecular Alterations Across Chemotherapeutic Agents

Chemotherapeutic agents induce multiple biochemical and molecular alterations that collectively contribute to neuronal dysfunction and cognitive impairment. A common feature shared by several agents is the induction of oxidative stress and mitochondrial dysfunction. Doxorubicin produces a pronounced pro-oxidant state, as reflected by depletion of reduced glutathione, elevated malondialdehyde (MDA) levels, and reduced antioxidant enzyme activity, indicating impaired redox balance [5]. Mitochondrial dysfunction, including impaired respiratory chain activity and altered mitochondrial morphology, has also been reported following doxorubicin administration [27]. Similarly, cisplatin induces mitochondrial dysfunction associated with enhanced oxidative stress, increased lipid peroxidation and nitrosative stress, elevated MDA and nitrite levels, and depletion of endogenous antioxidant defenses, including SOD, glutathione, and catalase [86,87]. Methotrexate likewise increases lipid peroxidation and decreases the GSH/GSSG ratio, indicating impaired redox homeostasis [88]. Cyclophosphamide, 5-FU, paclitaxel, and docetaxel also promote ROS generation and oxidative stress, accompanied by activation of pro-apoptotic pathways [70,85,89,90,91,92].

Neuroinflammation and apoptosis represent another major feature of chemotherapy-induced neurotoxicity. Doxorubicin increases the expression of inflammatory mediators, including IL-1β and NF-kB, together with pro-apoptotic markers such as Bax and caspase-3 [78]. Cisplatin similarly promotes activation of NF-kB and elevates IL-1β and TNF-α levels, while suppressing the Nrf2/HO-1 antioxidant signaling pathway [87]. Cyclophosphamide increases pro-inflammatory cytokines, including IL-6 and TNF-α, while reducing anti-inflammatory cytokines such as IL-4 and IL-10 [85,89]. Methotrexate dysregulates inflammatory mediators including IL-1β, TNF-α, COX-2, and iNOS [78,93]. Paclitaxel activates Toll-like receptor-mediated signaling, leading to NF-kB-dependent upregulation of pro-inflammatory cytokines [94], whereas docetaxel increases TNFα, NF-kB, and GFAP expression while activating p38α- and MAPK-dependent apoptotic signaling [91,92,95].

Chemotherapeutic agents also disrupt neurotrophic support, synaptic integrity, and neurotransmission. Cisplatin decreases expression of the presynaptic marker synaptophysin and the glutamatergic vesicular transporter Vglut2 [61,96], while reducing BDNF expression and impairing synaptic plasticity [87]. Cyclophosphamide, methotrexate, 5-FU, paclitaxel, and docetaxel similarly reduce neurotrophic factor expression [78,85,89,90,93,94,95]. Doxorubicin impairs glutamate clearance in the frontal cortex and dentate gyrus [97] and increases acetylcholinesterase activity while reducing hippocampal choline levels [5,27], indicating disturbances in glutamatergic and cholinergic neurotransmission. In addition, 5-FU decreases dopamine release in the striatum [90] and reduces synaptic protein expression, whereas docetaxel decreases expression of neuronal adhesion proteins such as NCAM [95].

Certain molecular alterations appear to be agent-specific. Cisplatin crosses the blood–brain barrier, at least in part via the copper transporter CTR1, allowing its accumulation within the CNS [98,99]. Similarly, 5-FU crosses the BBB via passive diffusion [100,101] and disrupts white matter integrity by reducing oligodendrocyte precursor synthesis and promoting demyelination, particularly in the corpus callosum [23]. These changes may contribute to reduced cerebral blood flow, impaired glucose metabolism, and behavioral abnormalities [102]. Paclitaxel additionally induces endoplasmic reticulum stress [103], whereas methotrexate decreases hippocampal neural precursor cell proliferation [78,93]. Collectively, these biochemical and molecular alterations compromise neuronal homeostasis, connectivity, and survival, providing a mechanistic basis for chemotherapy-induced cognitive impairment [1,78].

### 6.5. Behavioral and Cognitive Outcomes Across Chemotherapeutic Agents

Chemotherapeutic agents consistently induce behavioral and cognitive deficits across multiple domains in rodent models, including impairments in spatial memory, recognition memory, learning, working memory, attention, and exploratory behavior. Although the severity and persistence of these deficits vary among agents, the overall behavioral profile closely resembles the cognitive dysfunction observed in patients with chemotherapy-induced cognitive impairment [1].

Spatial learning and memory are among the most consistently affected cognitive domains. Doxorubicin-treated animals exhibit increased escape latency in the Morris water maze (MWM), indicating impaired hippocampal-dependent navigation [104], while persistent deficits in spatial learning have also been demonstrated in the Barnes maze (BM) several weeks after treatment [50]. Similarly, cisplatin-treated rats spend significantly less time in the target quadrant in the MWM [87], and cyclophosphamide-treated mice show impaired spatial memory and learning, reflected by reduced target quadrant exploration in the MWM and deficits in the Y-maze [85,105]. Methotrexate- and 5-FU-treated animals likewise demonstrate impairments in spatial memory and learning during the subacute phase, as evidenced by poorer performance in the BM, although partial recovery may occur at later time points [6]. Likewise, paclitaxel- and docetaxel-treated rodents also display impaired spatial learning and memory, including increased escape latency, reduced time spent in the target quadrant in the MWM, and deficits in maze-based behavioral paradigms [77,106,107,108,109,110,111].

Recognition memory is likewise impaired following exposure to several chemotherapeutic agents. Doxorubicin reduces exploration time and discrimination index in the novel object recognition (NOR) test [112] and impairs contextual fear conditioning and inhibitory avoidance learning [72]. Cisplatin similarly decreases preference for novel objects and reduces freezing behavior during contextual fear conditioning [87,113]. Cyclophosphamide disrupts passive avoidance learning and object recognition memory [114]. Docetaxel impairs performance in the NOR and object location task (OLT), resulting in reduced exploration of novel or displaced objects and impaired short-term memory [77,109,110,111]. In contrast, methotrexate- and 5-FU-treated animals show relatively preserved recognition memory in the NOR test despite deficits in other cognitive domains, suggesting that acute recognition memory may be less sensitive than spatial learning to chemotherapy-induced injury [6].

Attentional deficits and alterations in exploratory behavior have also been reported. Cisplatin administration reduces the percentage of correct responses and increases omissions in the 5CSRTT [96], whereas methotrexate and 5-FU impair performance in the 5CSRTT during the subacute phase [6]. Docetaxel also produces deficits in attention, working memory, and learning, as demonstrated by impaired performance in the simple chronic reaction time task (SCRTT) and other behavioral paradigms [77,109,110,111]. Doxorubicin decreases innate exploratory behavior, reflected by a reduced number of rearings in the open field test [115]. Collectively, these behavioral abnormalities are closely associated with the structural, biochemical, and molecular alterations described above, including neuroinflammation, oxidative stress, and impaired neuroplasticity (Table 1) [67,85,89,103,116,117].

### 6.6. Dose, Route of Administration and Model-Specific Notes for Individual Chemotherapeutic Agents

#### 6.6.1. Doxorubicin

In preclinical studies, doxorubicin is most commonly administered i.p. or i.v. in rodents, with dosing regimens selected to approximate clinically relevant exposures. Examples from the literature include: male Sprague-Dawley rats (2 mg/kg/week, i.p. for 4 weeks) [104,118], female Wistar rats (2.5 mg/kg, i.p., once every 5 days for 50 days) [112], male B6C3 mice (single 20 mg/kg, i.p.) [119], male Wistar rats receiving either 3.5 mg/kg/week i.p. for 8 weeks [120] or escalating single doses of 0.8, 2, and 8 mg/kg i.p. [115], and aged female mice (12–13 months) received doxorubicin via two i.p. injections of 5 mg/kg, resulting in a cumulative dose of 10 mg/kg [50]. These regimens allow for the assessment of cumulative neurotoxic effects, while variations in age, sex, and species emphasize the critical importance of careful model selection.

#### 6.6.2. Cisplatin

In preclinical studies, cisplatin is most frequently administered via intraperitoneal injection, with dosing schedules and cumulative exposure varying across species and experimental designs. For example, male C57BL76J mice were treated with 2.3 mg/kg i.p. following a 5-day injection–5-day no injection–5 day injection protocol [86,96], while male Wistar rats received 5 mg/kg/week i.p. for 5 weeks [121] or 7 weeks [87]. Other regimens included male Sprague-Dawley rats receiving 5 mg/kg/week for 4 weeks [113] or postnatal day 25 Sprague-Dawley rats receiving 2 mg/kg i.p. for 5 consecutive days [122]. These protocols illustrate the variability in experimental design and highlight that repeated or cumulative dosing tends to produce more pronounced neurotoxic and cognitive effects. Differences in species, age, and strain should therefore be carefully considered when interpreting cisplatin-induced neurotoxic and cognitive outcomes [1].

#### 6.6.3. Cyclophosphamide

In rodents, cyclophosphamide is typically administered i.p., with both single high doses and repeated lower doses used in different models. In mice, single i.p. doses typically range from 40 to 200 mg/kg [114,123,124], whereas rats are often treated with single doses around 100 mg/kg [125]. Repeated administration has also been employed in both species, with weekly or sustained dosing over several weeks generally involving lower individual doses. Sensitivity to cyclophosphamide varies across species and strains, with mice and rats showing differential susceptibility. Notably, repeated exposure or higher cumulative doses are more consistently linked to pronounced cognitive impairment and histopathological alterations [126,127].

#### 6.6.4. Methotrexate

In preclinical models, methotrexate is most often administered i.p. or i.v., with doses reported to produce cumulative neurotoxic effects. In one study, female Sprague Dawley rats (6–8 weeks old, 150–220 g) received a single intraperitoneal injection of 37.5 mg/kg once weekly for two consecutive weeks, with behavioral and molecular assessments performed 96 h, 31 days, and 93 days post-treatment [6]. Methotrexate was also administered at 250 mg/kg intraperitoneally in male Long Evans rats, using a repeated dosing schedule over two weeks with follow-up weekly injections [128]. Species-specific differences influence the severity and duration of cognitive impairments.

#### 6.6.5. 5-Fluorouracil

In preclinical models, 5-FU is commonly administered i.p. or i.v., with repeated dosing leading to cumulative neurotoxic effects. Sensitivity and severity vary among species and strains. In one study, female Sprague Dawley rats (6–8 weeks old, 150–220 g) were administered 5-FU via a single i.p. injection at 75 mg/kg once per week for two consecutive weeks. Outcomes were evaluated at 96 h, 31 days, and 93 days post-treatment [6]. In another study, female C57BL/J mice received 5-FU at 100 mg/kg, three times per week [129].

#### 6.6.6. Paclitaxel

In preclinical models, paclitaxel is typically administered i.p. or i.v., with repeated dosing producing cumulative neurotoxic effects. Sensitivity differs across species and strains, with some rodents exhibiting more pronounced cognitive deficits. Reported regimens in rodents include 2 mg/kg/day intraperitoneally for four days in male Sprague-Dawley rats (6–8 weeks old) [106], 20 mg/kg intraperitoneally for 12 injections over 4 weeks in male C57BL/6 mice (9 weeks old) [107], and acute (10 mg/kg/day intraperitoneally for 7 days) or chronic (10/mg/kg/day intraperitoneally for 30 days) treatment in male C57BL/6 mice [108]. In older male Sprague-Dawley rats (10 months old), 2 mg/kg intraperitoneally administered on four alternating days was used [1,82].

#### 6.6.7. Docetaxel

In preclinical models, docetaxel is most frequently administered i.p. or i.v., with dosing regimens varying widely depending on species, strain, and study design. Both single-dose and repeated-dose protocols have been employed to model acute and cumulative neurotoxic effects [1].

Intraperitoneal administration is most often used in rodent models, with reported doses ranging from low weekly regimens to high single dose exposures. In mice, docetaxel was administered i.p. at doses between approximately 8 and 33 mg/kg, either as a single injection or in a repeated weekly schedules extending up to four weeks [109,111]. In rats, i.p. dosing similarly included both single injections (e.g., 10–30 mg/kg) and short-term weekly treatment paradigms [1,77,95].

Intravenous administration was also employed in specific models, particularly in adult Wistar rats, using lower weekly doses (e.g., 1 mg/kg) over several weeks to better approximate clinical infusion protocols [110]. Experimental designs differ further with respect to animal age, strain, and treatment duration, underscoring substantial model-specific variability in docetaxel exposure (Table S1) [1].

## 7. Discussion

### 7.1. Combination Chemotherapy Regimens in Preclinical Models of Chemobrain

In addition to single-agent models, some preclinical studies have explored combination chemotherapy regimens, including cyclophosphamide combined with methotrexate and 5-fluorouracil [130,131,132], methotrexate with 5-fluorouracil [133,134,135], as well as docetaxel, doxorubicin and cyclophosphamide (TAC regimen) [67]. These multi-agent protocols more closely resemble clinical treatment strategies and may lead to cumulative or synergistic neurotoxic effects. However, such approaches remain underrepresented in preclinical research, where the majority of studies continue to rely on single-agent exposure, thereby limiting translational applicability.

Notably, these regimens are commonly used in clinical oncology, further emphasizing the need for their increased representation in experimental models.

### 7.2. Integrated Perspective on BBB Involvement and Comparative Mechanisms of Chemobrain

The blood–brain barrier (BBB) is a highly specialized, semi-permeable structure formed by brain microvascular endothelial cells, the basement membrane, pericytes, astrocytes, microglia, and neurons, collectively referred to as the neurovascular unit [136]. Its structural features, such as the presence of tight junctions, absence of fenestrations, and markedly reduced pinocytotic activity, restrict paracellular transport and ensure high selectivity [137]. Tight junction complexes are essential for maintaining barrier integrity and cellular polarity, with their disruption directly associated with increased permeability [136,138]. Pericytes, which cover a large proportion of cerebral capillaries, play a crucial role in regulating vascular stability, endothelial junction integrity, and cerebral blood flow [139]. Astrocytes further support BBB function through their perivascular end-feet, forming the glia limitans and contributing to ion homeostasis and metabolic waste clearance via the glymphatic system [140]. Functionally, the BBB tightly regulates the exchange of substances between the blood and the brain, allowing passive diffusion of small lipophilic molecules (<400–500 Da) and selective transport via specialized carriers, while preventing the entry of most macromolecules and over 98% of drugs into the central nervous system [138,141]. This highly coordinated structural and functional organization makes the BBB a key regulator of central nervous system homeostasis and a critical protective interface between the circulation and brain parenchyma.

**Figure 1:**
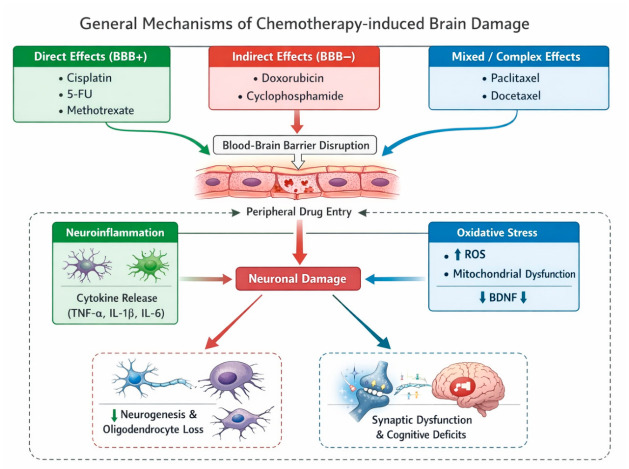
**Integrated mechanisms of chemotherapy-induced brain damage (CIBD).** Chemotherapeutic agents induce cognitive impairment through both direct and indirect pathways. Direct neurotoxic effects occur when drugs penetrate the blood–brain barrier (BBB), while indirect effects are mediated via peripheral inflammation, cytokine signaling, and BBB disruption. Within the central nervous system, interconnected processes including neuroinflammation, oxidative stress, mitochondrial dysfunction, impaired neurogenesis, synaptic alterations, and neurotransmitter dysregulation contribute to neuronal dysfunction. Crosstalk between these mechanisms amplifies brain injury, ultimately leading to deficits in memory, attention, and executive function.

Chemotherapeutic agents differ in their ability to interact with the BBB; however, their impact on the CNS ultimately results in neurotoxicity and cognitive damage. Cisplatin, one of the most famous neurotoxic chemotherapeutics, is capable of entering the CNS, partially via transport mechanisms such as CTR1, thereby inducing direct neuronal damage characterized by oxidative stress, mitochondrial dysfunction, and apoptotic signaling [98,99]. On the other hand, 5-fluorouracil crosses the BBB via passive diffusion and directly disrupts neurogenesis, microglia functioning, and white matter integrity [23,100,101]. Agents such as doxorubicin exhibit limited BBB penetration and primarily exert indirect neurotoxic effects through peripheral oxidative stress and cytokine release, including TNF-α, which can subsequently influence central inflammatory signaling [67]. Similar to this, cyclophosphamide [142] and methotrexate [71] also exert indirect neurotoxic effects through systemic inflammation, oxidative stress, and glial activation, although some effects may arise from different cytokine signaling and vascular alterations. Some chemotherapeutics, such as paclitaxel, have been shown to compromise BBB integrity by causing direct endothelial dysfunction and reducing microvascular density, thereby further increasing the CNS vulnerability to circulating inflammatory cytokines [79]. Despite the difference in their ability to penetrate BBB, chemotherapeutic agents activate different molecular pathways, including oxidative stress, neuroinflammation, apoptotic signaling, reduced neurotrophic support, and synaptic dysfunction, all of which are associated with memory impairment and diminished executive function (Figure 1).

### 7.3. Synthesis and Strength of Current Evidence

The interpretation of the available evidence is complicated by substantial heterogeneity across studies, including differences in experimental design, animal species and strains, dosing regimens, and routes of administration. As a result, the reported neurotoxic and cognitive outcomes are not uniformly consistent across models. While several mechanisms—such as oxidative stress, neuroinflammation, and BBB disruption—are recurrently described, their relative contribution and reproducibility vary considerably between studies. Therefore, generalized conclusions regarding uniformly negative effects of all chemotherapeutic agents should be interpreted with caution, taking into account the variability in methodological quality and experimental conditions.

Moreover, the strength of evidence differs between individual agents. Compounds such as doxorubicin and cisplatin are supported by a relatively robust body of experimental data, consistently demonstrating neurotoxic effects across multiple models and endpoints. In contrast, for agents such as taxanes and antimetabolites, the available data are more limited and, in some cases, yield mixed or context-dependent results. Consequently, certain mechanistic insights remain provisional and require further validation in well-standardized experimental settings. Subsequent studies should aim to improve comparability across models and to strengthen the evidence base for agents where current data remain sparse or inconclusive.

Studies using genetically engineered mouse (GEM) models [2,50] have provided additional insight into the mechanisms underlying chemotherapy-induced cognitive impairment, complementing findings from standard animal models.

## 8. Clinical Implications, Risk Factors and Future Perspectives

Although considerable progress has been made in understanding the mechanisms underlying chemotherapy-induced cognitive impairment, effective preventive and therapeutic strategies remain limited. Currently, no validated diagnostic biomarkers or disease-modifying treatments are available, and clinical management is largely based on symptomatic care, cognitive rehabilitation, lifestyle modification, and supportive interventions [143,144,145,146,147].

Additional supportive strategies, including cognitive-behavioral therapy and structured cognitive training, aim to improve patients’ coping strategies and quality of life despite persistent cognitive symptoms [144,145,146].

Among currently available supportive strategies, physical exercise represents one of the most promising non-pharmacological approaches for mitigating chemotherapy-associated cognitive changes. In experimental models, regular exercise enhances hippocampal neurogenesis, increases brain-derived neurotrophic factor (BDNF) expression, and improves cognitive performance following chemotherapy [80,148,149,150,151]. Likewise, environmental enrichment and cognitive stimulation promote neuronal plasticity and alleviate cognitive deficits in preclinical models [152,153,154,155].

Importantly, not all patients exposed to chemotherapy develop persistent cognitive impairment, suggesting that individual susceptibility plays a significant role. Current evidence indicates that genetic background, DNA repair capacity, immune regulation, and antioxidant defense mechanisms may influence the risk of chemotherapy-induced cognitive dysfunction. Polymorphisms in genes involved in neuronal repair and plasticity, including APOE, BDNF, and COMT, have been proposed as potential susceptibility factors, although their clinical significance remains to be fully established [156,157]. Likewise, chronic inflammation, oxidative stress, and persistent DNA damage may interact to amplify neuronal injury in vulnerable individuals, potentially contributing to long-term cognitive decline [7]. These findings highlight the importance of developing individualized risk assessment strategies that could facilitate early identification of patients at increased risk and support the implementation of personalized preventive and supportive interventions.

Several experimental pharmacological strategies targeting synaptic plasticity have also demonstrated encouraging results in preclinical models. Modulation of glutamatergic neurotransmission and calcium-dependent signaling pathways has been shown to improve dendritic complexity and attenuate cognitive deficits in rodent models of chemotherapy-induced cognitive impairment [110,158,159,160,161,162,163,164,165]. However, these approaches remain experimental, and their clinical efficacy and safety require further investigation before routine implementation.

Several pharmacological approaches have shown encouraging results in experimental studies. Antidepressants such as fluoxetine and the mood stabilizer lithium have been shown to enhance hippocampal neurogenesis and improve cognitive performance in rodent models of chemotherapy-induced cognitive impairment [3,100,101,107,166]. However, robust clinical evidence supporting the routine use of these agents for the prevention or treatment of chemotherapy-induced cognitive impairment is still lacking.

Neuroinflammation has also emerged as a promising therapeutic target. Several experimental approaches aimed at modulating microglial activation, astrocyte function, and myelin preservation and repair have shown beneficial effects in preclinical models of chemotherapy-induced cognitive impairment [69,74,167]. Nevertheless, currently available anti-inflammatory and immunomodulatory therapies have produced inconsistent clinical results and may be associated with significant adverse effects, highlighting the need for safer and more specific therapeutic strategies [168,169,170].

Future research should focus on identifying reliable biomarkers for early diagnosis, clarifying patient-specific risk factors, including potential genetic susceptibility, and conducting well-designed clinical trials evaluating both pharmacological and non-pharmacological preventive strategies [156,157]. In parallel, increasing awareness of chemotherapy-induced cognitive impairment among healthcare professionals, patients, and caregivers may facilitate earlier recognition of cognitive symptoms and timely implementation of supportive interventions. Ultimately, a better understanding of the cellular and molecular mechanisms underlying chemotherapy-induced brain injury will facilitate the development of personalized preventive and therapeutic strategies aimed at improving long-term neurological outcomes and quality of life in cancer survivors.

## 9. Conclusions

After reviewing the preclinical evidence on chemotherapy-induced brain damage, it is evident that multiple chemotherapeutic agents—including DNA-targeting drugs (e.g., doxorubicin and cisplatin), antimetabolites (e.g., methotrexate and 5-fluorouracil), and taxanes (e.g., paclitaxel and docetaxel)—induce multifaceted neurotoxic effects. While these drug classes share common mechanisms such as oxidative stress, DNA damage, apoptosis, and synaptic alterations, the severity, consistency, and persistence of cognitive impairments vary across agents and experimental conditions.

These alterations are reflected in histological, biochemical, and molecular changes in rodent models and are functionally manifested as deficits in learning, memory, attention, and executive function, paralleling the clinical phenomenon of chemobrain. Importantly, the current body of evidence reveals both well-established mechanisms and areas of inconsistency, particularly regarding long-term cognitive outcomes and the relative vulnerability of specific brain regions.

Future research should focus on improving the standardization of experimental models, conducting longitudinal studies to assess the persistence of cognitive deficits, and further elucidating molecular pathways that could serve as therapeutic targets. Addressing these gaps, along with the translational challenges discussed in this review, is essential for developing effective neuroprotective strategies and for improving the quality of life of cancer survivors. Overall, this review provides a focused and updated synthesis of preclinical evidence in rodent models, highlighting common and divergent mechanisms of chemotherapy-induced cognitive impairment and identifying key gaps for future research.

## Figures and Tables

**Table 1:** Neurotoxic, histological, biochemical and molecular effects of chemotherapeutic agents in rodent models (↑ indicates an increase).

Chemotherapeutic Agent	Mechanisms of Neurotoxicity	Histological Changes in Animal Models	Biochemical and Molecular Alterations	Behavioral Assessments
Doxorubicin	Oxidative stress Neuroinflammation Activation of apoptotic pathways DNA damage Reduced BDNF levels	Neuronal shrinkage and dendritic simplification Increased apoptosis in hippocampus and prefrontal cortex Astrocyte and microglial activation (↑ GFAP)	Oxidative stress and redox imbalance Mitochondrial dysfunction Apoptotic and inflammatory signaling Neurotransmitter dysregulation (glutamatergic and cholinergic)	Novel object recognition test Inhibitory avoidance task Open field test Morris water maze Barnes maze
Cisplatin	DNA crosslinking and genomic instability Mitochondrial dysfunction and oxidative stress Apoptotic signaling Glial activation	Hippocampal and cortical neuronal loss Reduced dendritic complexity Synaptic alterations Impaired neurogenesis in hippocampus, lateral ventricles and corpus callosum Gliosis	BBB penetration via copper transporter (CTR1) Oxidative and nitrosative stress with impaired antioxidant defenses Activation of NF-kB-mediated inflammatory pathways Synaptic protein downregulation Reduced neurotrophic support (BDNF)	5-choice serial reaction time task Morris water maze Novel object recognition task
Cyclophosphamide	DNA alkylation and genomic damage Oxidative stress and apoptosis Microglia-driven neuroinflammation	Neuronal loss in the hippocampus and prefrontal cortex Dendritic atrophy Synaptic alterations	Increased ROS, pro-apoptotic markers, and pro-inflammatory cytokines Reduced neurotrophic and anti-inflammatory signaling	Morris water maze Y-maze Passive avoidance test Novel object recognition test
Methotrexate	Disruption of folate metabolism Oxidative stress-mitochondrial dysfunction-apoptosis axis Neuroinflammation and gliosis Impaired cerebral blood flow Neurotransmitter imbalance Reduced BDNF expression	Neuronal loss and dendritic simplification (hippocampus, prefrontal cortex) White matter alterations Gliosis and neuroinflammation (↑ GFAP)	Oxidative stress and redox imbalance Pro-apoptotic and pro-inflammatory signaling Reduced neurotrophic support	Barnes maze 5-choice serial reaction time task Novel object recognition test
5-Fluorouracil	DNA damage and inhibition of thymidylate synthase Oxidative stress, mitochondrial dysfunction, and apoptosis Disruption of glial cell function Neuroinflammation with elevated hippocampal cytokines Reduced hippocampal BDNF expression	Neuronal loss and dendritic spine reduction (hippocampus, prefrontal cortex) White matter/myelin abnormalities Reduced proliferation of neural precursor cells Astrocyte activation with increased GFAP expression	Oxidative stress (↑ ROS, pro-apoptotic markers, inflammatory cytokines) Reduced neurotrophic support and synaptic protein expression Neurotransmitter imbalance White matter disruption and impaired oligodendrocyte precursor proliferation Reduced cerebral blood flow and glucose metabolism	Barnes maze 5-choice serial reaction time task Novel object recognition test
Paclitaxel	Microtubule stabilization and disrupted axonal transport Mitochondrial dysfunction, oxidative stress, and apoptosis Neuroinflammation and glial activation (↑ peripheral cytokines) Calcium signaling disruption Hippocampal endothelial senescence and reduced microvascular density Compromised BBB integrity	Axonal degeneration and dendritic spine loss Microtubule disorganization (hippocampus, cortex)	Oxidative stress and activation of pro-apoptotic pathways Endoplasmic reticulum stress Toll-like receptor-mediated signaling and NF-kB-dependent pro-inflammatory cytokine upregulation	Morris water maze Novel object recognition test
Docetaxel	Microtubule stabilization and disrupted axonal transport Mitochondrial dysfunction, oxidative stress and apoptosis Impaired glial function with hippocampal cytokine upregulation	Neuronal loss and dendritic spine reduction Microtubule disorganization (hippocampus, cortex)	Oxidative stress (↑ ROS; MDA) Activation of pro-apoptotic signaling pathways Calcium dysregulation and mitochondrial compromise Upregulation of inflammatory mediators Reduced neurotrophic support and antioxidant defenses	Morris water maze Novel object recognition test Object location task 5-choice serial reaction time task

## Data Availability

No new data were created or analyzed in this study. Data sharing is not applicable to this article.

## Supplementary Materials

The following supporting information can be downloaded at: https://www.mdpi.com/article/10.3390/brainsci16070750/s1, Table S1: Study level details of chemotherapy-induced neurotoxicity in rodents.
