# Supplementary material for: Chemotherapy-Induced Brain Damage: Mechanisms and Insights from Rodent Models
Source: Brain Sci. 2026 Jul 15;16(7):750. doi: 10.3390/brainsci16070750 (PMC13406774; doi:10.3390/brainsci16070750)
Supplement: Supplementary file 1 [file brainsci-16-00750-s001.zip › brainsci-4400377-supplementary.pdf]

Supplementary Table S1. Study level details of chemotherapy-induced neurotoxicity in rodents

Chemotherapeutic agent/ Reference	Species	Strain	Sex	Age/ Weight	Tumor status	Dose	Route	Treatment schedule	Time to testing	Behavioral/ Cognitive outcomes
**Doxorubicin [112]**	Rat	Wistar	Female	12 weeks	Tumor-free	2.5 mg/kg	i.p.	Once every 5 days for 50 days	After treatment	NOR - Impaired recognition/ episodic memory
**Doxorubicin [104]**	Rat	Sprague-Dawley	Male	6 weeks (210–230g)	Tumor-free	2 mg/kg/week	i.p.	Once a week for 4 week	After treatment	MWM Impaired spatial learning and memory (↑escape latency)
**Doxorubicin [120]**	Rat	Wistar	Male	6 weeks	Tumor-free	3.5 mg/kg/week,	i.p.	8 weeks	After treatment	Impaired learning and reduced exploratory behavior
**Doxorubicin [115]**	Rat	Wistar	Male	2-3 months	Tumor-free	Single increasing doses (0.8, 2 and 8 mg/kg)	i.p.	Single increasing doses (0.8, 2 and 8 mg/kg)	After treatment	Impaired learning and reduced exploratory behavior
**Doxorubicin [50]**	Mouse	C57BL/6J	Female	12-13 months	Tumor-free	5 mg/kg x 2	i.p.	Two injections	Weeks post-treatment	BM Persistent spatial learning deficits
**Cisplatin [96]**	Mouse	C57BL/6J	Male	5–6 months	Tumor-free	2.3 mg/kg/day	i.p.	2 cycles of daily injections followed by a 5-day rest without injections	After treatment	5-CSRTT Impaired attention (↓correct responses, ↑omissions)
**Cisplatin [87]**	Rat	Wistar	Male	5-6 weeks (150-200g)	Tumor-free	5 mg/kg/week	i.p.	7 weeks	After treatment	Impaired spatial and recognition memory
**Cisplatin [113]**	Rat	Sprague-Dawley	Male	200–250g	Tumor-free	5 mg/kg/week	i.p.	4 weeks	After treatment	Impaired learning and recognition memory (↓freezing, ↓discrimination)
**Cyclophosphamide [105]**	Mouse	Not specified	Not specified	18-25g	Tumor-free	100 mg/kg	i.p.	every 2 alterna-tive days (4 treatments)	After treatment	Impaired spatial and recognition memory
**Cyclophosphamide [114]**	Mouse	ICR	Male	8-10 weeks	Tumor-free	40 mg/kg	i.p.	Single dose	After treatment	Impaired learning and recognition memory

**Methotrexate [6]**	Rat	Sprague-Dawley	Female	6-8 weeks (150-220g)	Tumor-free	37.5 mg/kg	i.p.	Once per week, 2 consecutive weeks 96h, 31d, 93d	After treatment	Deficits in spatial memory, working memory, attention, learning (31 days) Partial recovery at 93 days NOR relatively preserved
**Methotrexate [128]**	Rat	Long-Evans	Male	10 weeks	Tumor-free	250 mg/kg	i.p.	Repeated dosing over 2 weeks + weekly follow ups	After treatment	Spatial memory deficits
**5-Fluorouracil/ [6]**	Rat	Sprague-Dawley	Female	6-8 weeks (150-220g)	Tumor-free	75 mg/kg	i.p.	Once per week, 2 consecutive weeks 96h, 31d, 93d	After treatment	Impairments in BM, 5CSRTT; hippocampal-dependent deficits persist; NOR largely unaffected
**5-Fluorouracil/ [129]**	Mouse	C57BL/J	Female	8 weeks	Tumor-free	100 mg/kg	i.p.	3x/week over 3 weeks for a total of three injections (days 1, 8, and 15	After treatment	Deficits across spatial memory, working memory, attention, learning
**Paclitaxel [106]**	Rat	Sprague-Dawley	Male	6-8 weeks	Tumor-free	2 mg/kg/day	i.p.	4 days	During/ shortly after treatment	Spatial memory ↓ (MWM: ↑escape latency, ↓target quadrant time)
**Paclitaxel [107]**	Mouse	C57BL/6J	Male	9 weeks	Tumor-free	20 mg/kg	i.p.	4 weeks (12 injections)	After treatment	Memory and learning deficits (MWM/NOR)
**Paclitaxel [108]**	Mouse	C57BL/6J	Male	Adult	Tumor-free	10 mg/kg/day	i.p.	7 days	After treatment	Cognitive impairment
**Paclitaxel [108]**	Mouse	C57BL/6J	Male	Adult	Tumor-free	10 mg/kg/day	i.p.	30 days	After treatment	Cognitive impairment
**Docetaxel/ [109]**	Mouse	CD1	Male	27-33g	Tumor-free	8 mg/kg	i.p.	Single or weekly up to 4 weeks on days 0,9,18, and 28	After treatment	Impaired spatial and recognition memory
**Docetaxel [111]**	Mouse	C57BL/6J	Male	11 weeks	Tumor-free	33 mg/kg	i.p.	Single injection	After treatment	Cognitive, short-term memory and attention deficits

**Docetaxel [110]**	Rat	Han Wistar	Male	4-5 months	Tumor-free	1 mg/kg	i.v.	Once per week, for 4 weeks	After treatment	Spatial memory deficits and reduced exploration
